# Errors in Clinician–Parent Communication during Neonatal Hospitalization

**DOI:** 10.3390/children6060080

**Published:** 2019-06-24

**Authors:** John M. Idso, Mir A. Basir

**Affiliations:** Section of Neonatology, Department of Pediatrics, Medical College of Wisconsin, Milwaukee, WI 53226, USA; jidso@mcw.edu

**Keywords:** neonatology, communication, medical error, electronic medical record, family

## Abstract

Error in clinician–parent communication is not a new issue in pediatrics. It has been the impetus behind national initiatives, namely, family-centered rounds. While family-centered rounds have proven effective, their success is dependent on the family being present during rounds. This does not always occur during prolonged hospitalizations, particularly in the Neonatal Intensive Care Unit (NICU). Current communication methods with parents not present during rounds rely heavily on the provider’s prerogative and ability to multitask. Thus, errors in communication are commonplace and are largely accepted as inevitable. For the sake of the parents of a patient in the NICU, a high-fidelity communication system is urgently needed. NICUs must move beyond the telephone and use modern innovations in communication technology.

## 1. Introduction

“Feeder grower, nothing to do.” It was the Sunday night before my first shift on my acting internship in the Neonatal Intensive Care Unit (NICU), and my eyes scanned the sign out. One noticeably shorter description stood out among the rest, like an unfinished line of music in an orchestral score. I was not sure what a “feeder grower” was, but based on the brevity, I assumed this baby was relatively healthy compared to the others on the service, many of whom had several paragraphs of information. As I learned later, there is a cohort of premature babies at each NICU who have progressed past the many hurdles they face in their first weeks of life. The last milestones for these babies before they go home is to demonstrate they can eat enough without a feeding tube to grow on their own. These babies are colloquially referred to as “feeder growers” and require minimal medical decision-making. Appropriately, these babies require very little from the medical team, accounting for the short sign out. Although it may not seem possible in a patient whose only medical intervention was time, an error occurred during our month caring for this patient.

The baby, who we will call Dawson, was now gaining weight with his feeding tube removed. On rounds, our team decided on an appropriate discharge date for later that week and then promptly moved on to the next patient. Notably, Dawson’s mother was not present during rounds as she maintained a full-time job. The next morning when I walked into Dawson’s room during my usual preparation for rounds, I was greeted by a pinched expression on Dawson’s mother’s face. It was like the dam inside her that had been holding back the emotions of watching her newborn live in the hospital had suddenly cracked. She had learned of the planned discharge date from the night nurse, she told me, and she was shocked to learn Dawson would be coming home so soon, because no one from the team had updated her on his rapid progress towards discharge. I saw waves of fear, helplessness, and anger surface in the way she wrung her hands, held her shoulders, and shook as she spoke. Though most parents are delighted to learn their newborn is healthy enough to leave the hospital, she explained she felt blindsided by the news. I listened to her concerns, apologized on behalf of the team, and started the process of informing the team and making amends. The medical and nursing teams responded quickly, and after several conversations with Dawson’s team, Dawson’s mother switched from anger to relief as she had an opportunity to agree on the discharge date and felt empowered and informed. After he had spent 60 days in the NICU, I saw Dawson carried out of the hospital by his beaming mom on my last day of the rotation.

## 2. Discussion

The Institute of Medicine defines medical error as “the failure to complete a planned action as intended or the use of a wrong plan to achieve an aim” [1]. It is related to, but distinct from, an adverse event which is defined as “an injury caused by medical management rather than by the underlying disease or condition of the patient” [2]. 

How did this happen in spite of our meticulous attention to detail? Each day, I or one of the residents would review Dawson’s chart looking for changes in vital signs. We would calculate his Calories per kilogram per day, total fluid intake, and overnight weight change. We presented this data on rounds with the attending dietitian and nurse. With all of this time and expertise spent on Dawson each and every day, how did this adverse event occur? As I reflect on this experience, I cannot help but think that we were, in some ways, treating only one patient. We were so focused on medical perfection with the biomedical patient, Dawson, that we forgot about the patient surrogate, the parent.

Effective communication with families is not a new problem in pediatrics, and the NICU is no exception [3,4,5]. Family-centered rounds have been revolutionary in parent communication in pediatrics [6]. They are successful in achieving rapport, information sharing, and shared decision-making; however, many parents with children in the NICU are unable to attend rounds due to obligations to their work or other children. With current family leave laws, many parents feel that they have insufficient time off at home with their child due to extended NICU stays [7]. Thus, significant family leave reform is urgently needed to allow parents with children in the NICU protected time to support their child’s care while in the NICU and sufficient time after discharge to be successful in the transition home. Even with significant family leave reform, it cannot be assumed that all parents would choose to take extended time off from their careers [8].

Thus, for parents who are unable to attend rounds, communication with families occurs primarily at the discretion of the medical provider. This system, which relies upon the provider’s prerogative to communicate with the patient surrogate, leads to medical errors. A communication system that acts apart from a provider’s ability to prioritize and multitask is urgently needed to prevent future communication errors similar to Dawson’s case. The urgency to fill this communication gap is markedly increased, given the lagging cultural and political acceptance of sufficient family leave. These medical errors occur every day and add unnecessary stress on what is for many parents the most stressful time in their life [9].

The current generation of pediatric trainees is more adept in communication technology than any of its predecessors. It is time that we move beyond the telephone and usher in new communication norms. Our concerns for patient and physician privacy, while crucially important, cannot paralyze us from applying the innovations of our time to this problem. Our patient surrogates, the parents, deserve something better. I challenge us as patient advocates to creatively consider ways to implement modern communication modes to communicate daily with our patient surrogates who are not able to attend rounds.

Potential solutions exist along a continuum of automaticity and information curation. Examples of solutions that focus on automaticity include patient access to the medical chart [10,11,12] or automatic notification of discrete fields documented in the electronic medical record, such as weight or diaper contents. The advantage of automaticity is fast communication speed and low staff labor. The primary disadvantage of automaticity is that the information given may be too sophisticated or inappropriate for the parent.

Solutions necessitating information curation include secure apps that facilitate efficient and easy-to-understand communication between hospital staff and parents or telemedicine during rounds [13]. Many child care facilities are already using mobile phone applications to send regular updates. The advantage of highly curated information is that it is easy to understand. The disadvantage of highly curated information is that it can increase demands on hospital staff and is less likely to be timely. Additionally, telemedicine may not be an option for parents who are at work.

In order to attain the best blend of automaticity and information curation, I recommend a working group consisting of, but not limited to, parents, physicians, nurses, communication technology experts, and health informatics specialists. Importantly, the ideal communication method will vary depending on a variety of local factors such as parent education level, technology availability, and preexisting healthcare culture. As such, it is reasonable to predict that NICUs will offer several options for parents depending on their locality, education level, and comfort with technology. Each institution should form its own working group to most accurately assess its local needs. However, as electronic medical record (EMR) platforms are currently highly consolidated, a nationally organized group and consensus will be more likely to result in new EMR functionalities.

## 3. Conclusions

As a pediatric trainee, I have been striving to acquire the requisite medical knowledge and clinical acumen in order to give my patients the best chance at living a healthy life. Another way to phrase this goal is to provide care that implements the fruits of research without medical error. This experience was a wakeup call. Up until this point, I had been primarily focused on providing error-free care to the biomedical patient. While addressing the concerns and questions of the family has certainly been a part of my pediatric education, it has never been elevated to a level of importance similar to error-free patient care. I propose that errors with the patient surrogate be treated with a similar aversion as errors with the biomedical patient. We need to pursue excellence in providing care to both our patients: a child and their family.
